# Gut Microbiota in NSAID Enteropathy: New Insights From Inside

**DOI:** 10.3389/fcimb.2021.679396

**Published:** 2021-07-06

**Authors:** Xianglu Wang, Qiang Tang, Huiqin Hou, Wanru Zhang, Mengfan Li, Danfeng Chen, Yu Gu, Bangmao Wang, Jingli Hou, Yangping Liu, Hailong Cao

**Affiliations:** ^1^ Department of Gastroenterology and Hepatology, General Hospital, Tianjin Medical University, Tianjin Institute of Digestive Diseases, Tianjin Key Laboratory of Digestive Diseases, Tianjin, China; ^2^ Tianjin Key Laboratory on Technologies Enabling Development of Clinical Therapeutics and Diagnostics, School of Pharmacy, Tianjin Medical University, Tianjin, China

**Keywords:** non-steroidal anti-inflammatory drug enteropathy, gut microbiota, toll-like receptor 4, proton pump inhibitors, probiotics, fecal microbiota transplantation

## Abstract

As a class of the commonly used drugs in clinical practice, non-steroidal anti-inflammatory drugs (NSAIDs) can cause a series of adverse events including gastrointestinal injuries. Besides upper gastrointestinal injuries, NSAID enteropathy also attracts attention with the introduction of capsule endoscopy and double balloon enteroscopy. However, the pathogenesis of NSAID enteropathy remains to be entirely clarified. Growing evidence from basic and clinical studies presents that gut microbiota is a critical factor in NSAID enteropathy progress. We have reviewed the recent data about the interplay between gut microbiota dysbiosis and NSAID enteropathy. The chronic medication of NSAIDs could change the composition of the intestinal bacteria and aggravate bile acids cytotoxicity. Meanwhile, NSAIDs impair the intestinal barrier by inhibiting cyclooxygenase and destroying mitochondria. Subsequently, intestinal bacteria translocate into the mucosa, and then lipopolysaccharide released from gut microbiota combines to Toll-like receptor 4 and induce excessive production of nitric oxide and pro-inflammatory cytokines. Intestinal injuries present in the condition of intestinal inflammation and oxidative stress. In this paper, we also have reviewed the possible strategies of regulating gut microbiota for the management of NSAID enteropathy, including antibiotics, probiotics, prebiotics, mucosal protective agents, and fecal microbiota transplant, and we emphasized the adverse effects of proton pump inhibitors on NSAID enteropathy. Therefore, this review will provide new insights into a better understanding of gut microbiota in NSAID enteropathy.

## Introduction

Non-steroidal anti-inflammatory drugs are widely used in osteoarthritis, rheumatoid arthritis, fevers, and various pain symptoms for their anti-inflammatory, analgesic, and antipyretic functions (Rosenbloom and Craven, 1983). It is reported that there are 30 million people worldwide taking NSAIDs every day, and NSAIDs have a prescription of 111 million per year in the United States, valued at approximately 480 million dollars (Laine, 2001). NSAIDs play a therapeutic role by inhibiting cyclooxygenase and reducing the synthesis of prostaglandins (Bacchi et al., 2012). However, toxicities to the gastrointestinal, cardiovascular, and renal systems are recorded in the use of various kinds of NSAIDs such as aspirin, naproxen, and indomethacin, of which, gastrointestinal side effects are the most dangerous due to its high incidence and severity (Utzeri and Usai, 2017; Radi and Khan, 2019). For instance, approximately 1,000,000 hospitalizations happened per annum due to serious gastrointestinal complications of NSAIDs, furthermore, there were 16,500 NSAIDs-related deaths in among patients with rheumatoid arthritis or osteoarthritis (Wolfe et al., 1999). The upper gastrointestinal symptoms caused by NSAIDs are mainly peptic ulcer, bleeding, perforation, etc. But NSAIDs-induced damage mainly concentrates in the small intestine, distal to the ligament of Treitz, especially the ileum (Aabakken and Osnes, 1989), and the main manifestations include inflammation, increased permeability, bleeding, ulcers, and malabsorption (Lanas and Sopeña, 2009). Importantly, conventional test methods such as gastroscopy and colonoscopy are difficult to make a clear diagnosis of the NSAID enteropathy. Therefore, it has been neglected for a long time, and its pathogenesis has not yet been fully elucidated. Recently, growing clinicians found that NSAIDs frequently injure the small bowel due to the introduction of new technologies such as capsule endoscopy and balloon-assisted endoscopy (Graham et al., 2005). Although PG deficiency is recognized as an important pathogenic factor of NSAIDs-induced gastrointestinal injuries, gut microbiota also contributes to NSAID enteropathy. Meanwhile, proton pump inhibitors (PPIs) and bile participate in the development of NSAID enteropathy (Barrios and Lichtenberger, 2000; Chen et al., 2014). In this review, the relationship between gut microbiota and NSAID enteropathy, and the therapy that target gut microbiota are summarized, with a focus on recent data.

## NSAID Enteropathy: A Neglected Disease

The GI tract side effects of NSAIDs are mainly manifested in NSAIDs-induced gastropathy, and NSAID enteropathy (Sinha et al., 2013; Svistunov et al., 2018). The incidence of NSAIDs-related gastric and duodenal injuries is 9–22% (Cann, 1990). With the use of capsule endoscopy and balloon-assisted endoscopy, more attention has been focused on NSAID enteropathy. Graham et al. revealed that 71% of the patients with arthritis had small intestine injury after taking non-selective NSAIDs for more than 3 months whereas only 10% of non-NSAIDs users had small intestine injury, and the incidence of bleeding caused by NSAID enteropathy is higher than that of NSAIDs-induced gastropathy (Graham et al., 2005; McCarthy, 2009). Meanwhile, compared to non-selective NSAIDs, selective COX-2 inhibitors were proven to have better gastroduodenal safety profiles, for instance, the 2-week supplement with celecoxib caused fewer small intestinal injury than that with naproxen (Goldstein et al., 2005). Furthermore, in a randomized, double-blinded trial, intestinal safety of lumiracoxib was also proven in healthy volunteers (Hawkey et al., 2008). However, compared to healthy volunteers, it was reported that the chronic treatment of selective COX-2 inhibitors caused small-bowel injuries, and there was no obvious difference with non-selective NSAIDs (Maiden et al., 2007).

Among long-term NSAID users, the intestinal injuries including inflammation, mucosal erosion, increased mucosal permeability, and ulceration, and more serious clinical outcomes such as perforation and diverticulitis have been described (Lanas and Sopeña, 2009). Meanwhile, Concentric diaphragmatic strictures were a secondary complication of chronic use of NSAIDs (Bjarnason et al., 1988), which were considered as a unique and rare characteristic of NSAID enteropathy, and it was related to chronic inflammatory status causing of scarring and fibrosis. Maiden et al. reported that only 2% had diaphragm-like strictures in 120 patients on long-term NSAIDs by capsule endoscopy (Maiden et al., 2007). The damages described above could cause clinical symptoms such as anemia, symptoms of stricture (i.e., abdominal distension, abdominal pain, constipation, and recurrent vomiting), hypoalbuminemia, and occult bleeding that may develop into iron-deficiency anemia (Morris et al., 1992; Smale et al., 2001; Scarpignato and Hunt, 2010).

Consequently, a simple and non-invasive detection method is necessary in the clinical field. Calprotectin is a biomarker of intestinal disorders, especially inflammatory diseases of GI tract (Ayling and Kok, 2018), and was used to diagnose NSAID enteropathy (Tibble et al., 1999). However, no significant correlation between fecal calprotectin and intestinal damage was also reported in some studies (Maiden et al., 2005; Goldstein et al., 2007), therefore, calprotectin is only used as a screening method. In addition, capsule endoscopy could directly observe the small intestine, some investigators evaluated the severity of NSAID enteropathy by using the Lewis score, which is a capsule endoscopic grading system for small bowel mucosal inflammation (Gralnek et al., 2008), and inflammatory status was divided into three groups: normal or clinically insignificant change, mild mucosal inflammatory change, and moderate or severe change. But the sensitivity and specificity of capsule endoscopy were 83.3 and 95.8% (Tachecí et al., 2010), and it is expensive and time-consuming, which limited the widely clinical use. As for treatment, discontinuing the NSAIDs or reducing the NSAIDs dose is necessary, switching to selective COX-2 inhibitors is also a common choice. Rebamipide and misoprostol are often used for NSAID enteropathy, which were reported to reduce the incidence of small intestinal lesions induced by diclofenac (Niwa et al., 2008; Fujimori et al., 2009). Sulfasalazine, a disease-modifying antirheumatic drug, could reduce blood loss in patients receiving NSAIDs (Hayllar et al., 1994), so it was used for enteropathy prevention in rheumatoid patients. And metronidazole and rifaximin are effective treatment for NSAID enteropathy (Bjarnason et al., 1992; Scarpignato et al., 2017). However, there is presently no way to extremely effective prevention and treatment of NSAID enteropathy.

## The Gut Microbiota: Composition and Functions

The gut microbiota is composed of a large and diverse community of microorganisms, including bacteria, viruses, fungi, archaea, and parasites, which play a critical role in the global health of the host (Fan and Pedersen, 2021). Sender et al. reported that the total number of gut bacteria in the 70kg “reference man” to be 3.8 × 10^13^, which is the same order as the number of human cells (Sender et al., 2016). Furthermore, the majority of the gut microbiota reside in the colon with estimates of about 1,011 bacteria/ml, compared to the concentration of bacteria in the stomach, duodenum, and jejunum is only 10^3^–10^4^ bacteria/ml (Sender et al., 2016). And there are distinct microbial habitats along the stomach, small intestine, and colon in the mammalian lower gastrointestinal tract. Chemical and nutrient gradients are changed along the gastrointestinal tract as well as host immune activity, which affects microbial community composition. At the genera level, *Prevotella*, *Streptococcus*, *Veillonella*, *Rothia*, and *Haemophilus* are normally inhabited in the healthy human stomach due to its acid-resistance (Nardone and Compare, 2015). In addition, *Firmicutes* and *Actinobacteria* are the dominant phyla in the duodenum where has high levels of bile acids, pancreatic secretions, antimicrobials agents, and oxygen (El Aidy et al., 2015). The jejunum is suitable for the growth of Gram-positive aerobes and facultative anaerobes such as including *Lactobacilli*, *Enterococci*, and *Streptococci* (El Aidy et al., 2015). And there are anaerobes and Gram-negative organisms in the distal part of ileum which is close to the ileocecal valve, and Villmones et al. reported that *Streptococcus*, *Granulicatella*, *Actinomyces*, *Solobacterium*, *Rothia*, *Gemella*, and *TM7(G-1)* were most frequently detected in the distal ileum (Villmones et al., 2018). Finally, the colon is mainly dominated by *Firmicutes* and *Bacteroidetes* (Eckburg et al., 2005).

The genome sequences of gut microbiome cover 3 × 10^6^ genes, which are roughly 150 times the number of the human genome (Qin et al., 2010). Despite the microorganisms colonized in the digestive tract are significantly distinctive among people and depend on host specificity, functional gene profiles are quite similar (Lozupone et al., 2012). In a healthy state, gut microbiota can promote food digestion and lipid and glucose metabolism, participate in immune function, and synthesize amino acids and vitamins required by the human body (Heintz-Buschart and Wilmes, 2018). When the host is immersed in pathogenic factors, the composition of gut microbiota may change subsequently, which promotes the development of several diseases such as colorectal cancer, inflammatory bowel disease, osteoarthritis, and hypertension (Abreu and Peek, 2014; Jackson et al., 2018; Yang et al., 2018). Besides, the gut microbiome can also alter bioavailability, bioactivity, and toxicity by transforming the drug’s structure whereas many non-antibiotic drugs, such as PPIs and metformin change microbiome composition and function (Forslund et al., 2015; Freedberg et al., 2015). Some studies found that the exposure of the NSAIDs in the small bowel was increased by bacterial β-glucuronidase, then lead to NSAID enteropathy (Boelsterli and Ramirez-Alcantara, 2011; Lucas, 2016).

## Alternations of Gut Microbiota in NSAID Enteropathy

Robert A et al. first reported that GF rats were resistant to indomethacin-induced small intestinal damage in 1977 (Robert and Asano, 1977), and the following evidence supported the potential role of gut microbiota in NSAID enteropathy (Xiao et al., 2017; Yoshikawa et al., 2017; Maseda and Ricciotti, 2020) (**Table 1**).

**Table 1:** The changes of the gut microbiota in subjects receiving NSAIDs.

Authors	Subjects	Type of NSAIDs	Treatmentperiod	Sample	The changes of the gut microbiota	
Edogawa et al. (2018)	Male (n = 11) Female (n = 12)	Indomethacin (75 mg twice daily)	5 days	Faeces	**↑** *Prevotellaceae* (female)	**↑** *Firmicutes* (male)
					**↑** *Ruminococcus* (male)	**↑** *Bacteroidetes* (female)
					**↓** *Firmicutes* (female)	**↓** *Ruminococcus* (female)
			5 days	Duodenal aspirates	**↓** *Proteobacteria* (male)	**↓** *Pseudomonadaceae* (male)
					**↓** *Proteobacteria* (male)	**↓** *Alphaproteobacteria* (male)
					**↓** *Proteobacteria* (male)	**↓** *Rhizobiales* (male)
Bokulich et al. (2016)	Obese post-menopausal women (n = 10)	Celecoxib (200 mg twice daily)	10 days	Feces	No significant abundance alterations	
Rogers et al. (2016)	Adults (n = 155)	Celecoxib	>30 days	Feces	**↑** *Acidaminococcaceae*	**↑** *Enterobacteriaceae*
		Ibuprofen	>30 days	Feces	**↑** *Propionibacteriaceae*	**↑** *Pseudomonadaceae*
					**↑** *Puniceicoccaceae*	**↑** *Rikenellaceae*
		Multiple NSAIDs	>30 days	Feces	**↑** *Acidaminococcaceae*	**↑** *Desulfovibrionaceae*
					**↑** *Enterococcaceae*	**↑** *Erysipelotrichaceae*
		NSAIDs and PPIs	>30 days	Feces	↑*Bacteroides*	**↑** *Erysipelotrichaceae*
Mäkivuokko et al. (2010)	Elderly people (n = 9)	Multiple NSAIDs (three or more times weekly)	3 weeks	Feces	**↓** *Roseburia*	**↓** *Ruminococcus*
					**↓** *Collinsella* spp.	**↓** *Lactobacillus*
Yoshihara et al. (2020)	Mice (male)	Aspirin (200 mg/kg) Omeprazole (20 mg/kg)	3 h 9 weeks	Jejunum	**↑** *Akkermansia*	**↓** *Bifidobacterium*
Colucci et al. (2018)	Rat (male)	Diclofenac (4 mg/kg BID)	14 days	Ileum	**↑** *Proteobacteria*	**↑** *Bacteroidetes*
Xiao et al. (2017)	Mice	Indomethacin (10 mg/kg)	24 h	Feces	**↑** *Firmicutes*	**↓** *Bacteroides*
Blackler et al. (2015a)	Rat (male)	Naproxen (20 mg/kg)	2 days	Jejunum	**↑** *Bacteroidaceae*	**↑** *Porphyromonadaceae*
					**↑** *Enterococcaceae*	**↓** *Lachnospiraceae*
					**↑** *Enterobacteriaceae*	
		Naproxen PPIs	2 days 9 days	Jejunum	**↑** *Pseudomonadaceae*	**↑** *Enterobacteriaceae*
Liang et al. (2015)	Mice (male)	Indomethacin (10 mg/kg)	24 h	Luminal contents	**↑** *Peptococcaceae*	**↑** *Erysipelotrichaceae*
			6 h	Feces	**↓** *Bacteroidetes*	**↑** *Firmicutes*
					**↑** *Ruminococcus*	**↑** *rc4-4*
					**↑** *Lachnospiraceae*	**↑** *Anaeroplasma*
Dalby et al. (2006)	Rat (female)	Indomethacin (7.5 mg/kg)	48 h	Lymph nodes	**↓** *Staphylococcus* spp.	**↓** *Streptococcus* spp.
					**↑** *Lactobacillus* spp.	**↑** *Enterococcus faecalis* spp.
					**↑** *Clostridium difficile* spp.	
Uejima et al. (1996)	Rat (male)	BFMeT (1,000 mg/kg)	72 h	Ileum	**↑** *Escherichia coli*	**↑** *Klebsiella*
					**↑** *Proteus*	**↑** *Bacteroides*

NSAIDs, non-steroidal anti-inflammatory drugs; BFMeT, 5-bromo-2-(4-fluorophenyl)-3-(4-methylsulfonylphenyl) thiophene; PPIs, Proton pump inhibitors.

Rogers et al. found that aspirin could raise the abundance of *Prevotella*, *Bacteroides*, *Ruminococcaceae*, and *Barnesiella* while the abundance of *Acidaminococcaceae* and *Enterobacteriaceae* was increased because of the treatment of celecoxib and ibuprofen. And the increased abundance of bacteria from families *Propionibacteriaceae*, *Puniceicoccaceae*, *Pseudomonadaceae*, and *Rikenellaceae* was observed in individuals taking ibuprofen, compared to either non-users or naproxen users (Rogers and Aronoff, 2016). But in a longitudinal study, the composition of the gut microbiota in post-menopausal obese women was not changed by celecoxib (Bokulich et al., 2016), this result may be related to the individual variability in response to celecoxib. Furthermore, Mäkivuokko et al. reported that the abundance of microbes in older was less than younger subjects, but the total number of the gut microbiota is higher in older taking NSAIDs compared with youngers and non-users. However, the abundance of *Collinsella*, *Actinobacteria*, and *Lactobacilli* was reduced in fecal microbiota composition of older subjects using NSAIDs (Mäkivuokko et al., 2010). And in acute intestinal injuries induced by indomethacin, fecal and duodenal microbiota diversity was decreased especially in women, meanwhile, the increased abundance of *Actinobacteria phylum* and reduced abundance of *Bacteroidetes* and *Proteobacteria* was presented in women compared to men (Edogawa et al., 2018). These results reminded that the impact of NSAIDs on the composition of human gut microbiota may be influenced by age, sex, NSAIDs type, diet, and psychological stress and so on.

Small intestinal bacterial overgrowth (SIBO) is defined as the presence of bacteria ≥10^5^ colony-forming units per milliliter in the culture of the upper gut aspirate, which represents small intestinal flora dysbiosis (Ghoshal and Ghoshal, 2017). Motoko et al. evaluated the connection between SIBO and severity of NSAIDs-induced small intestinal damage by using the lactulose hydrogen breath test (LHBT) in patients who had used NSAIDs for more than 3 months. And LHBT-positive patients were obviously associated with severe NSAIDs-induced small intestinal damage (Muraki et al., 2014). However, more research needs to be conducted to confirm a clear association between SIBO and NSAID enteropathy.

Compared to fewer data about the human intestinal microbiota, there are more animal studies. Gram-positive bacteria reside in the small intestine under physiological conditions and will be replaced by the Gram-negative bacteria such as *Proteobacteria* and *Bacteroidetes* in rats after NSAIDs administration (Uejima et al., 1996; Colucci et al., 2018). After the ulcers presented in rats, multiple intestinal bacteria dominated by Gram-negative and anaerobic bacterial species quickly colonized the ulcers (Elliott et al., 1998), thereby delaying the healing of the ulcers. Those species such as *Enterococcus faecalis*, *Clostridium*, *Bacteroides*, and *Escherichia coli* were universally grown in the intestinal ulcers (Dalby et al., 2006). And Sara et al. reported that ileal enterococcal colony-forming units were significantly increased after administration of indomethacin (Mayo et al., 2016). And more studies supported that the *Escherichia coli* and *Enterococcus* spp. were increased in NSAID enteropathy (Lanas and Scarpignato, 2006; Montalto et al., 2010). Interestingly, Uejima et al. showed that gnotobiotic rats mono-associated with *Eubacterium limosum* or *Escherichia coli* had numerous ulcers in the small intestine whereas gnotobiotic rats mono-associated with *Bifidobacterium* or *Lactobacillus* had no ulcers (Uejima et al., 1996). And indomethacin was reported to cause intestinal inflammation and increase the proportion of *Bacteroides* and *Enterobacteriaceae* in the ileum and cecum-colon and *Clostridium* in the ileum of male rats (Terán-Ventura et al., 2014). In addition, diet and psychological stress could also aggravate progress of NSAID enteropathy. It was reported that high-fat diet induced gut microbiota dysbiosis exacerbated small intestinal damage in mice treated with indomethacin, accompanied by a reduced abundance of *Bifidobacteriaceae*, and *Streptococcaceae* and an increased abundance of *Erysipelotrichaceae*, and *Ruminococcaceae* in the HFD group (Sugimura et al., 2019). And Yoshikawa et al. uncovered that psychological stress increased intestinal permeability and exacerbated NSAID enteropathy in mice by enhancing the proportion of Gram-negative bacteria (Yoshikawa et al., 2017). Permpalung et al. reported that the risk of *Clostridium difficile*-associated diarrhea was increased among patients exposed to NSAIDs (Permpalung et al., 2016), and indomethacin can increase severity of *Clostridium difficile* infection in mouse model (Muñoz-Miralles et al., 2018). Further research showed that NSAIDs alter the gut microbiota and exacerbate *Clostridium difficile* colitis (Maseda et al., 2019). These results revealed that NSAIDs could cause *Clostridium difficile* infection, which exacerbates intestinal damage by releasing two protein exotoxins (TcdA and TcdB) (Leffler and Lamont, 2015). Since the small intestinal bacteria are difficult to sample, there were few studies about specific bacteria in NSAID enteropathy, and further research is required.

This data indicated that Gram-negative species and anaerobic species contribute to the development of NSAID enteropathy. Gram-negative bacteria thrive and accumulate in the small intestine, producing endotoxins and acids after NSAIDs supplement, which results in increased intestinal permeability and bacterial translocation, and further aggravate the intestinal injury.

## Mechanisms Which Gut Microbiota Contributing to NSAID Enteropathy

**Figure 1:**
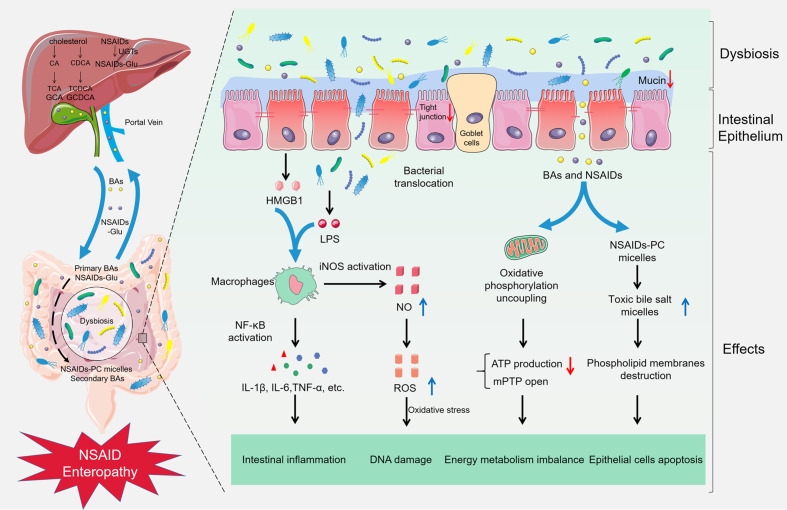
Gut microbiota in NSAID enteropathy. The supplement of NSAIDs could lead to the gut barrier destruction, intestinal dysbiosis, and bacterial translocation. Subsequently, LPS from the Gram-negative bacteria and HMGB1 from the damaged epithelial cells combine with TLR-4, resulting in activation of NF-κB through the MyD88-dependent pathway, increasing the production of cytokines such as TNF-α, IL-1β, and IL-6, subsequently inducing intestinal inflammation in NSAID enteropathy. Furthermore, the activation of NF-κB also can up-regulate the expression of iNOS and produce excessive NO, finally damage the DNA and enzymes of epithelial cells in oxidative stress. Meanwhile, NSAIDs can cause an uncoupling of oxidative phosphorylation in the mitochondria and reduce ATP production and open the mitochondrial permeability transition pore (mPTP), which leading to energy metabolism imbalance. Besides, NSAIDs can competitively bind to phosphatidylcholine (PC) with bile acids, and increase the formation of toxic bile salt micelles, destroy the phospholipid membrane, and contribute to the pathogenesis of NSAID enteropathy.

The pathogenesis of small intestine damage caused by NSAIDs is complex. First, NSAIDs play a pharmacological role by inhibiting cyclooxygenase and reducing the production of prostaglandin. Nevertheless, prostaglandin contributes to the mucosal defense system in the gut (Takeuchi and Satoh, 2015). Next, most NSAIDs can lead to uncoupling of oxidative phosphorylation in mitochondria and deplete the inner transmembrane potential of mitochondria (Mahmud et al., 1996). It will reduce adenosine triphosphate production and open the mitochondrial permeability transition pore, thus causing energy metabolism imbalance, followed by enterocyte death and the gut barrier destruction (Somasundaram et al., 1997). And then, growing evidence suggests that gut microbiota participates in NSAID enteropathy progression, the administration of NSAIDs will disrupt the balance of gut microbiota, which results in the proliferation of Gram-negative bacteria and the reduction of Gram-positive species. Subsequently, pathogenic bacteria activate inflammatory pathway by Toll-Like Receptor 4 and release inflammatory factors. Microbiota disorder also improves the toxicity of bile acid and aggravates the NSAID enteropathy. Meanwhile, the gut microbiota participates in the intestinal injuries induced by PPIs and NSAIDs (**Figure 1**).

### Role of Toll-Like Receptor 4

It has been reported that gut microbiota participates in the development of NSAID enteropathy through Toll-Like Receptor (TLR) 4-dependent pathway (Watanabe et al., 2008). The TLR family is one of the members of the transmembrane pattern recognition receptors (PRRs) family, and it is responsible for recognizing pathogen-associated molecular patterns (PAMPs) or damage-associated molecular patterns (DAMPs) such as bacteria and viruses, triggering subsequently dendritic cell maturation, inflammatory and antiviral responses, thus eliminating the invading pathogens. So, it plays a critical role in innate immune responses against microbial pathogens, and the subsequent induction of adaptive immune responses as well (Brennan and Gilmore, 2018). Different TLRs recognize different molecular patterns of microorganisms and self-components. For example, TLR4 mainly recognizes lipopolysaccharides (LPS), a membrane component originated from the Gram-negative bacteria, resulting in activation of nuclear factor-κB (NF-κB) through the MyD88-dependent pathway (Kuzmich et al., 2017; Chen et al., 2018). Subsequently it led to the increased production of cytokines such as tumor necrosis factor- α (TNF-α), interleukin (IL)-6, or IL-12, and induced leukocyte recruitment in NSAID enteropathy (Fujiyama et al., 2007; Takeuchi and Akira, 2010). With the suppression of TNF-α and monocyte chemotactic protein-1, the small intestinal damage induced by indomethacin has been decreased in TLR4^–/–^ mice and MyD88^–/–^ mice (Watanabe et al., 2008). Nadatani et al. revealed that high mobility group box 1 (HMGB1) releasing from damaged cells could combine to TLR-4 and activate NF-κB through the MyD88-dependent pathway in NSAID enteropathy (Nadatani et al., 2012).

The nucleotide-binding and oligomerization domain-like receptors (NLRs) are also members of the PRRs family and can recognize PAMPs resulting in the formation of the inflammasome. The inflammasome promotes the processing of prointerleukin-1β into mature IL-1β (Otani et al., 2017), and the transcription of pro-IL-1β is induced by the activation of NF-κB (Latz et al., 2013). NLRP3 protein belongs to the family of NLRs and is called “pyrin domain-containing protein 3.” NLRP3 inflammasome is one of the most investigated molecules, because NLRP3 recognizes many molecules, including PAMPs and DAMPs, and a variety of molecules closely associated with diseases (Lamkanfi and Dixit, 2012). So, it is associated with sundry diseases, such as metabolic disorders, multiple sclerosis, and inflammatory bowel disease, etc. (Shao et al., 2015). Higashimori et al. reported that mature IL-1β as an important proinflammatory cytokine induced by NLRP3 inflammasome was involved in the pathophysiology of NSAID enteropathy (Higashimori et al., 2016). Meanwhile, colchicine can prevent NSAID enteropathy by inhibiting the activation of NLRP3 inflammasome (Otani et al., 2016). All these data highlighted the role of NLRP3 inflammasome in NSAID enteropathy.

LPS also up-regulated the expression of inducible nitric oxide synthase (iNOS) and caused the increased nitric oxide (NO) production (Boughton-Smith et al., 1993; Takeuchi et al., 2010), and indomethacin and diclofenac can also inhibit mitochondrial complex I activity in Caco-2 cells, eventually resulting in increased reactive oxygen species (ROS), which could damage the DNA of epithelial cells as a strong oxidant (Banan et al., 2000; Potoka et al., 2002). In addition, growing evidence has shown that endoplasmic reticulum (ER) stress participates in NSAID-induced cell death (Ock et al., 2017; Chang et al., 2020), and ER stress could induce NLRP3-mediated inflammation and cell death (Lerner et al., 2012). Studies have demonstrated that NSAIDs could induce apoptosis of glioma cells and Caco-2 cells by inducing ER stress (Boonyong et al., 2020; Chang et al., 2020), and high levels of ROS activate the unfolded-protein response to lead to inflammatory response (Chaudhari et al., 2014), meanwhile, ER stress which exacerbated by oxidative stress can destroy the morphology and function of mitochondrial (Malhotra and Kaufman, 2007). These results may reveal the possible mechanism of ER stress in the development of NSAID enteropathy. Some pathogenic bacteria are described to induce ER stress and unfolded-protein response. *Helicobacter pylori* induces ER stress by vacuolating cytotoxin and subsequently causes mitochondrial damage and apoptosis in gastric epithelial cells (Akazawa et al., 2013). And another pathogen, *Listeria monocytogenes* can activate the unfolded-protein response *via* secretion of cytolysin listeriolysin O (Pillich et al., 2012). However, the interplay between gut microbiota and ER stress in NSAID enteropathy is rarely reported so far, and further studies are needed.

### Enterohepatic Circulation of NSAIDs and Bile Acids

Studies have implicated that bile acids (BAs) seem to have a pivotal role in the development of NSAID enteropathy, since the ligation of the bile duct can prevent small intestinal damage caused by NSAIDs in rats (Jacob et al., 2007). Primary bile acids are synthesized in the liver, and subsequently combined to glycine or taurine to create conjugated primary bile acids (Joyce and Gahan, 2016). Afterward, the conjugated bile acids are secreted into the bile and reserved in the gallbladder. About 95% of bile acids will be reabsorbed in the ileum and recycled by the liver, which is enterohepatic circulation (Trauner and Boyer, 2003). And the primary bile acids are excreted into the small bowel and catalyzed into the free secondary bile acids by bile salt hydrolases, which are refined by the gut microbiota such as *B. fragilis*, *Clostridium perfringens*, *Listeria monocytogenes*, *Bacteroides vulgatus*, *Lactobacillus*, and *Bifidobacterium*, etc. (Jones et al., 2008; Gérard, 2013). The pathogenic roles of bile acids in intestinal injury were presented in both *in vivo* and *in vitro* studies. The intestinal inflammation was increased after administration of ursodeoxycholic acid in rats taking indomethacin (Uchida et al., 1997), and deoxycholic acid and taurodeoxycholate could produce pro-inflammatory cytokine such as IL-8, and then activate NF-κB in HT29 and IEC-6 cells (Strauch et al., 2003). Interestingly Henri et al. found that in GF mice, secondary bile acids are rarely discovered in fecal samples, compared to a higher proportion of conjugated bile acids (Duboc et al., 2013). This confirms the irreplaceable role of the microbiota in the conversion of the bile acids. Meanwhile, in GF mice, the resistance of small intestinal damage induced by indomethacin is higher than in normal mice (Robert and Asano, 1977). Thus, the supplement of NSAIDs may lead to gut microbiota disorder and increase the production of the secondary bile acids (Hofmann, 1999; Martinez-Augustin and Sanchez de Medina, 2008), consequently, aggravate the NSAID enteropathy. However, in a new study, Lázár et al. found that NSAIDs increased conjugated bile acids in ileum, but not bile hydrophobicity, meanwhile, mucosal injury was correlated negatively with free bile acids and Gram-positive bacteria, and positively with conjugated bile acids and Gram-negative species (Lázár et al., 2021). These results showed that the increased bile hydrophobicity did not contribute to indomethacin-induced small intestinal damage and revealed the complex mechanism of hydrophobic secondary bile acids in NSAID enteropathy.

Simultaneously, the enterohepatic circulation of NSAIDs contributes to the development of intestinal damage (Kent et al., 1969). NSAIDs are absorbed in the stomach owing to their lipophilicity (McCormack and Brune, 1987). Subsequently, NSAIDs that contain carboxylic acids, are transported to the liver *via* the portal system after their administration either orally or intraperitoneally. And then, they are converted to NSAIDs-Glu by combining with glucuronic acid, taurine, or sulfate and excreted into the bile under the action of uridine diphosphoglucuronosyltransferase (UGTs) (Peris-Ribera et al., 1991; Mohri et al., 1998; King et al., 2001). Bile is drained into the gut after diet, subsequently, the small intestine is immersed in the NSAIDs and its oxidative conjugated metabolites. Then glucuronide is decomposed by the bacterial β-glucuronidase and generates aglycones when bile reaches the ileum (Boelsterli and Ramirez-Alcantara, 2011), and NSAIDs are transferred again into the enterohepatic circulation. As a result, the gut is exposed to NSAIDs and bile acids with a high concentration for a long time. Inhibitor-1, an inhibitor of bacterial β-glucuronidase, was revealed to prevent NSAID enteropathy in mice (LoGuidice et al., 2012), and ciprofloxacin alleviates NSAID enteropathy in rats by inhibiting intestinal β-glucuronidase activity (Zhong et al., 2016). These results showed the important role of bacterial β-glucuronidase in the development of NSAID enteropathy. Moreover, NSAIDs could competitively bind to phosphatidylcholine with bile acids, resulting in the formation of more toxic bile salt micelles, thereby destroying the phospholipid membrane (Barrios and Lichtenberger, 2000), and contribute to the pathogenesis of NSAID enteropathy (Mayo et al., 2016). Therefore, in combination with bile acids, NSAIDs aggravate the small intestinal injury due to the enterohepatic circulation.

### The Influence of Proton Pump Inhibitors

As mentioned above, peptic ulcers are the main manifestation in NSAIDs-induced gastropathy, happen in approximately 15–30% of chronic NSAIDs users (Laine, 1996; Singh et al., 2006; Lanza et al., 2009). Co-prescription of PPIs has been recommended to prevent NSAIDs-induced gastropathy in the current clinical guidelines (American College of Rheumatology Ad Hoc Group on Use of Selective and Nonselective Nonsteroidal Antiinflammatory Drugs, 2008; Lanza et al., 2009). As a result of the combination of PPIs and selective cyclooxygenase 2 inhibitors, hospitalizations who suffered upper GI tract side effects had drastically declined during the 1990s and 2000s. However, at the same time, hospitalizations rose significantly due to lower GI tract adverse events (Lanas et al., 2009). Consequently, PPIs may contribute to the development of NSAIDs enteropathy. Wallace et al. showed that PPIs dramatically aggravate distal intestinal damage of rodents, although they improve gastroduodenal damage because of the suppression of acid secretion (Wallace et al., 2011). Moreover, PPIs and histamine-2 receptor antagonists worsen the severity of intestinal injuries in rheumatoid arthritis patients who taking NSAIDs (Watanabe et al., 2013). Meanwhile, in a controlled and randomized trial, PPIs increased the proportion of subjects from 16.7 to 44.4% which developed NSAIDs-induced small intestinal injury, and the number of erosion in the intestine was also escalated in PPIs groups using capsule endoscopy (Washio et al., 2016).

The mechanism linking the intestinal injury to the PPIs is thought to be the gut microbiota disorder. One study elucidated that after the administration of PPIs, the numbers of *Enterococcus*, *Streptococcus*, *Staphylococcus*, and potentially pathogenic species *Escherichia coli* was increased significantly in 211 participants (Imhann et al., 2016). Naproxen enhanced the cytotoxic effects of the bile, and the toxicity was higher in rats co-treated with naproxen and PPIs (Blackler et al., 2015a). Furthermore, Blackler et al. reported that omeprazole or lansoprazole could increase the abundance of *γ-Proteobacteria*, including the families *Pseudomonadaceae* and *Enterobacteriaceae* in rats (Blackler et al., 2015a). Meanwhile, omeprazole significantly decreased *Actinobacteria*, whereas lansoprazole increased the relative abundance of *Bacteroidetes*. Therefore, PPIs could disrupt the gut microbiota and aggravate intestinal injury induced by NSAIDs. For example, high fructose diet, aspirin, and omeprazole can significantly reduce the *Bifidobacterium* and increase the *A. muciniphila* in the mouse jejunum and reduce the thickness of the jejunum mucus layer and jejunum goblet cells, thereby promoting the damage of the small intestine. Meanwhile, the administration of *Bifidobacterium bifidum G9-1* could inhibit the growth of *A. muciniphil*, restore the jejunum mucus layer and the number of goblet cells, and lead to the improvement of the NSAID enteropathy. These results may reveal the essential role of gut microbiota in intestinal injuries induced by NSAIDs and PPIs (Yoshihara et al., 2020). However, Yoda et al. reported that lansoprazole protected against indomethacin-induced small intestinal injuries in rats by inhibition of iNOS expression and up-regulation of heme oxygenase-1producton (Yoda et al., 2010), and revaprazan, a potassium-competitive acid blocker prevented NSAID enteropathy in rats through enhancing tight junction related mechanisms (Han et al., 2020), which presented a complicated influence of PPIs in NSAID enteropathy.

### Microbiota Metabolites in NSAID Enteropathy

The gut microbiota-derived metabolites include bile acids, short-chain fatty acids (SCFAs), branched-chain amino acids, trimethylamine N-oxide, tryptophan, and indole derivatives, and maintain intestinal integrity by regulating mucosal immune homeostasis and energy metabolism in health condition, however, they will participate in the pathogenesis of some metabolic disorders and gastrointestinal disease by destroying the intestinal barrier function and immune system under the circumstance of gut microbiota dysbiosis (Rooks and Garrett, 2016; Agus et al., 2021). Besides bile acids, SCFAs were reported to be related to NSAID enteropathy, long-term use of NSAIDs can obviously decrease the concentrations of SCFAs, such as isobutyrate, isovaleriate, and L-lactate in the elderly, meanwhile the proportion of acetate, propionate, and butyrate were also reduced although not statistically significant, thereby destroying the normal energy supply of intestinal epithelial cells (Tiihonen et al., 2008). In addition, SCFAs can protect gut barrier, for example, butyrate, acetate, and propionate activate NLRP3 inflammasome to release IL-18 and thus improve gut barrier integrity (Macia et al., 2015). Therefore, the shortage of SCFAs maybe aggravates NSAID enteropathy.

Moreover, serotonin (5-hydroxytryptamine [5-HT]) affects intestinal secretion, peristalsis and motility, vasodilatation, and the absorption of nutrients, as an important gastrointestinal signaling molecule and neurotransmitter (Agus et al., 2018). The production of 5-HT is controlled by gut microbiota, 5-HT production in the colon and concentrations in the blood was impaired in GF mice (Yano et al., 2015), and some SCFAs and secondary bile acids can also stimulate enterochromaffin cells to release 5-HT (Reigstad et al., 2015; Yano et al., 2015). The collaborate of serotonin-selective reuptake inhibitors and NSAIDs will aggravate upper gastrointestinal bleeding implicated the potential role of 5-HT in NSAID enteropathy (Anglin et al., 2014). It was reported that the serotonin was increased in NSAID-treated mice (Whitfield-Cargile et al., 2016), and the increased serotonin could enhance the intestinal motility which could deteriorate small bowel injuries induced by NSAIDs (Satoh et al., 2009; Agus et al., 2018). The specific mechanism which serotonin was increased by NSAIDs is not yet fully clear, Akiba et al. proposed that NSAID-induced SIBO generated excess SCFAs and promoted serotonin released from enterochromaffin cells *via* free fatty acid receptor 2 (Akiba et al., 2017). These observations showed the potential role of microbiota metabolites in the progress of NSAID enteropathy. However, tryptophan-metabolite indole which is lavish in the healthy human gut, decreased intestinal inflammation and injury in mice treated with indomethacin, and prevented the increase of *Bacteroidales* and instead resulted in an increase of *Clostridiales* (Whitfield-Cargile et al., 2016), this result maybe bring a new therapy for NSAID enteropathy.

## Possible Treatment Strategies in NSAID Enteropathy

### Antibiotics

Studies suggested that Gram-negative bacteria increased in the intestine after NSAIDs administration (Kinouchi et al., 1998; Wallace et al., 2011; Liang et al., 2015), and the increase of bacterial endotoxin significantly contributed to ulceration in the intestine (Watanabe et al., 2008). It is reasonable to suppose that antibiotics could reduce the Gram-negative species which might improve NSAID enteropathy. Koga et al. reported that kanamycin can prevent the indomethacin-induced small intestinal injuries dose-dependently in rats by decreasing the number of Gram-negative bacteria and endotoxin concentration of the small intestine (Koga et al., 1999). Rifaximin, a poorly absorbed antibiotic with a broad spectrum of antibacterial activity, could significantly improve NSAIDs-induced intestinal damage through counterbalancing the increase in *Proteobacteria* and *Firmicutes* abundance induced by indomethacin and decreasing tissue inflammation and oxidative stress (Fornai et al., 2016; Colucci et al., 2018). Metronidazole was able to kill Gram-negative bacteria and Gram-positive anaerobes and was effective in improving indomethacin-induced enteropathy in the result of the direct protective effect on uncoupling of mitochondrial oxidative phosphorylation caused by NSAIDs (Leite et al., 2001). Besides, there were many studies presenting that a cocktail of antibiotics also plays a positive role in protecting against NSAID enteropathy (Kent et al., 1969; Uejima et al., 1996; Kinouchi et al., 1998). A cocktail of antibiotics including neomycin, polymixin B, and bacitracin can reduce the mortality and severity of intestinal ulcers caused by indomethacin (Kent et al., 1969), as well as ampicillin, vancomycin, neomycin, and metronidazole (Syer et al., 2015).

These observations highlight the positive influence of antibiotics on the NSAID enteropathy. However, antibiotics are not consistently effective in some studies (Kinouchi et al., 1998). Syer SD et al. reported that treatment with kanamycin, vancomycin, and rifaximin failed to improve the severity of enteropathy which was induced by the naproxen in rats (Syer et al., 2015). Even, Xiao et al. documented that gut microbiota depletion *via* antibiotics including neomycin, polymycin, and metronidazole after administration of indomethacin could lead to an increase in mortality rate in mice (Xiao et al., 2017), this result may be relative to the depletion of probiotics and highlight the difference of NSAIDs. Meanwhile, the long-term use of antibiotics may improve multi-drug resistance of the gut microbiota and induce microbial disorders, leading to the occurrence of multiple intestinal diseases such as irritable bowel syndrome. Therefore, the clinical application of antibiotics is limited by the above problems.

### Probiotics and Prebiotics

It has been documented that probiotics and prebiotics had a potential therapeutic effect in inflammatory bowel disease and irritable bowel syndrome (Segarra et al., 2016; Ishaque et al., 2018; Ballini et al., 2019). And the supplement of *Bifidobacteria* enriched commensal bacteria can lead to the retrieval of the intestinal injury during treatment with omeprazole and naproxen by the restoration of *Actinobacteria* numbers in rats (Wallace et al., 2011), Kinouchi et al. confirmed that 72 h after administration of NSAIDs, the percentage of Gram-negative bacteria rose from 1.5 to 49.8%, but the percentage of Gram-negative rods was 9.7 or 16% in rats gavage culture supernatants of *L. acidophilus* or *B. adolescentis*, and the induction of ileal ulcers was decreased (Kinouchi et al., 1998). Besides, *Bifidobacterium breve Bif195* can reduce the severity of small intestinal damage induced by acetylsalicylic acid in a randomized, double-blind trial of healthy volunteers (Mortensen et al., 2019). Montalto et al. reported that treatment with a probiotics mixture that contained *Lactobacilli*, *Bifidobacteria*, and *Streptococcus salivaris subspecies thermophilus* for 21 days could reduce fecal calprotectin concentration in indomethacin-treated healthy volunteers compared to that in the placebo-treated volunteers (Montalto et al., 2010). In addition, there are only few studies on prebiotics in NSAID enteropathy, it has been reported that prebiotic lactoferrin alone prevented diclofenac-induced enteric damage so far (Fornai et al., 2020). And soluble dietary fibers, especially pectin, guar gum, and polydextrose can protect against NSAIDs-induced small intestine in cats (Satoh et al., 2010). These data highlight the beneficial effects of probiotics and prebiotics in experimental models of NSAID enteropathy (Guslandi, 2012).

In addition, probiotics and their metabolites maybe prevent NSAID enteropathy in different ways. *Lactobacillus casei strain Shirota* (LcS), a strain of lactic acid bacteria originally separated from humans and animals (Matsuzaki, 1998), could prevent the NSAID enteropathy (Watanabe et al., 2009). Meanwhile, both LcS culture supernatant and L-lactic acid could retrain NF-κB activation and increase TNF-α production in LPS-treated human monocytic cell line. These results showed that LcS has a preventive effect against NSAID enteropathy mediated by L -lactic acid. Syer SD et al. revealed that *B. longum subsp. longum NCC 1205* could produce acetate, while its mutant was unable to produce any acetate, however, both could obviously decrease the severity of the small intestinal ulceration caused by naproxen in rats (Syer et al., 2015). These observations suggested that acetate was not the potential mechanism for the protective effects of some probiotics such as *Bifidobacterium* and *Lactobacillus* species. Therefore, the potential mechanism of probiotics in NSAID enteropathy needs further study.

### Mucosal Protective Agents

It is well known that PG deficiency in the mucosa is an important causative factor in GI side effects induced by NSAIDs. It has been proposed that increasing the levels of mucosal PGs may improve the NSAID enteropathy. Misoprostol, a PGE1 derivative, was effective to prevent GI damage induced by NSAIDs including gastric damage and enteropathy (Aadland et al., 1987; Bjarnason et al., 1989). Watanabe et al. documented the fact that misoprostol could decrease the number of erosion and ulcer in the small intestine in patients treated with low-dose aspirin and PPIs (Watanabe et al., 2008). Moreover, misoprostol significantly prevented intestinal damage caused by diclofenac in healthy male volunteers (Fujimori et al., 2009). Since misoprostol tends to induce diarrhea at full therapeutic doses and the therapeutic effect in patients taking NSAIDs for a long-term has not been experimentally confirmed, it is not recommended for NSAID enteropathy at present.

Rebamipide is a mucosal protective drug that has been clinically used in treatment of gastritis and peptic ulcer (Arakawa et al., 1998), it can improve gastric defense by increasing PG production, increasing gastric mucosal blood flow, and reducing free oxygen radicals (Arakawa et al., 1998; Iijima et al., 2009). Rebamipide has been reported to prevent the gastroduodenal mucosa damage in patients taking NSAIDs (Park et al., 2007). Besides, Niwa et al. showed that rebamipide decreased the small intestinal lesions induced by 7-day treatment with diclofenac and PPIs compared to the placebo (Niwa et al., 2008). This was further confirmed in a larger-scale study which evaluated the protective effect of rebamipide in 80 healthy volunteers treated with 2 weeks diclofenac (Fujimori et al., 2011). The protective effect may be related to the regulation of gut microbiota. Tanigawa et al. reported that in the rebamipide-treated mice, *Lactobacillales* increased while the abundance of *Bacteroides* and *Clostridium subcluster XIVa* decreased (Tanigawa et al., 2013). And it was reported that the administration of rebamipide decreased the concentration of *Lactobacillus taiwanensis* whereas increased the proportion of *Lactobacillus murinus* (Tanigawa et al., 2021). Meanwhile, increased expression of α-defensin 5, a critical anti-microbial peptide produced by Paneth cells was found in rebamipide-treated mice (Ouellette, 2004). Therefore, rebamipide could inhibit NSAID enteropathy by upregulating α-defensin 5 and regulating gut microbiota.

In addition to misoprostol and rebamipide, tepretone is also a common mucosal protective agent, which can promote gastric mucus secretion, cell regeneration, and increased gastric blood flow (Terano et al., 1986; Shirakabe et al., 1995). And there were some studies reported that tepretone is effective for protecting against intestinal mucosal injuries induced by administration of diclofenac (Chitapanarux et al., 2019; Zhao et al., 2019). Thence, mucosal protective agents could be a candidate for protecting patients on long-term NSAID therapy.

### Fecal Microbial Transplantation

Fecal microbial transplantation (FMT) is a series of operations that gut microbiota from healthy donors is transplanted to sick patients through the upper or lower gastrointestinal route to recover intestinal microbial diversity, and is an effective therapy for many diseases such as *Clostridium difficile* infection and inflammatory bowel disease (Bouri and Hart, 2018; Chen et al., 2019). Interestingly, mice transplanted with feces from indomethacin-treated mice showed less intestinal injury and lower levels of proinflammatory cytokines when exposed to the NSAIDs, compared to the mice transplanted with feces from control groups (Xiao et al., 2017), and this result may be related to the adaptive beneficial changes in the gut microbiota. And Tanigawa et al. reported that the transplantation of the small intestinal microbiota of the rebamipide-treated mice improved the small intestinal damage induced by indomethacin and omeprazole (Tanigawa et al., 2021). This observation suggested the potential role of FMT in the treatment of the NSAID enteropathy. But another study recorded that the increased susceptibility to NSAIDs and increased intestinal permeability was transferable *via* cecal microbiota transplantation (Yoshikawa et al., 2017). Therefore, choosing the right gut microbiota in FMT may be a critical factor and the effect of FMT in NSAID enteropathy also needs further studies to be confirmed.

### Hydrogen Sulfide (H_2_S) Donor and NO Donor

Hydrogen sulfide (H_2_S) and NO, as endogenous gas signal molecule, play an important role in various of physiological and pathological processes, such as vasodilation, neural signaling, and oxidative stress (Panthi et al., 2016; Panthi and Gautam, 2017). H_2_S was reported to be effective for NSAID enteropathy, the administration of H_2_S-releasing naproxen derivative (ATB-346) could attenuate the gastrointestinal toxicity of naproxen in Wistar rats (Magierowski et al., 2017) due to its vasoactive properties and antioxidant effect (Wallace et al., 2010; Wallace et al., 2015). In addition, a protective dose of DADS, which is a H_2_S donor derived from garlic, could reverse cecal dysbiosis in rats induced by naproxen, and reduce the abundance of *Ruminococcaceae, Eubacteriaceae*, and *Enterococcaceae* (Blackler et al., 2015b). These observations revealed that H_2_S may protect NSAID enteropathy by regulating the gut microbiota. Furthermore, Porras et al. reported that NO donor LA-419 could decrease the expression of NO by inhibiting iNOS, subsequently prevent intestinal injuries in a rat model of enteritis induced by indomethacin (Porras et al., 2008). And NO could alleviate indomethacin-induced intestinal lesions in rats by preventing bacterial translocation, increasing mucus secretions, and inhibiting intestinal hypermotility. NO-donating NSAIDs is newly cyclooxygenase-inhibitors with reduced gastrointestinal toxicity (Whittle, 2003), and more research was confirming that NO-donating NSAIDs could bring less gastrointestinal injury compared to traditional NSAIDs (Fiorucci et al., 2007; Zhang et al., 2007). Overall, H_2_S or NO donor drugs may prove to be a new choice to ameliorate NSAID enteropathy.

### Other Promising Treatment for NSAID Enteropathy

Long-term use of NSAIDs always happens in patients with rheumatoid arthritis, and sulfasalazine is also commonly used in rheumatoid arthritis. It was reported that sulfasalazine could reduce blood loss induced by NSAIDs by detection of fecal excretion of 111indium labeled leukocytes and 51Chromium labeled red blood cell (Bjarnason et al., 1990; Hayllar et al., 1994). Furthermore, even with the combined use of PPIs, the application of mesalazine for 4 weeks could also decrease the small intestinal mucosal lesions caused by naproxen (Rácz et al., 2013). These results showed the protective effect of 5-aminosalicylic acid in NSAID enteropathy.

It was reported that less severe intestinal damage was existed induced by indomethacin in TNF-α-deficient mice (Fukumoto et al., 2011), and Watanabe et al. found that blocking of TNF-a by neutralizing antibodies can obviously protect against the small intestinal damage caused by indomethacin (Watanabe et al., 2008). In addition, a clinical study demonstrated anti-TNF therapy decreased the risk of NSAID enteropathy in rheumatoid arthritis patients (Watanabe et al., 2014). Therefore, anti-TNF therapy could be used in the treatment of NSAID enteropathy in the future.

Diet also protects against NSAID enteropathy, for example, Yanaka et al. found that sulforaphane, a substance rich in broccoli sprouts could reduce aspirin-induced intestinal damage in rats by activate Nrf2-Keap1 dependent antioxidant system and preventing anaerobic bacteria translocation to mucosal (Yanaka et al., 2013). And glutamate promoted mucus secretion and restrained bacterial invasion and iNOS expression to prevent small intestinal damage induced by loxoprofen, meanwhile, it promoted a healing of lesions by stimulation of VEGF expression and angiogenesis (Amagase et al., 2012). Besides, pasteurized chicken egg powder could stimulate the proliferation and migration of AGS, RIE1, and Caco-2 cells and decrease the severity of NSAID enteropathy (Playford et al., 2020).

## Conclusion

Despite NSAID enteropathy is getting more attention, the strategy for prevention and treatment for NSAID enteropathy is not established, and further research is required to elaborate on the mechanisms of the damage induced by NSAIDs. Although accumulating evidence suggests that gut microbiota contributes to the development of NSAID enteropathy, the interplay between NSAIDs and microbiota is not entirely expounded. In the present review, we summarized the potential role of the gut microbiota in the pathology of NSAID enteropathy or with the cooperation with PPIs, as well as the contribution of bile acids and microbiota-derived metabolites to this disease. Meanwhile, the preventive and therapeutic effect of antibiotics, probiotics, mucosal protective drugs, and FMT that can modulate the composition of intestinal flora is concluded. These results highlight the modulation of intestinal microbiota could be a new therapeutic strategy for such damage. Growing evidence from clinical and experimental studies suggests the specific gut microbiota is associated with diseases, and there is some disease-specific microbiota that can indicate specific diseases including inflammatory bowel diseases, colorectal cancer, and obesity. The metagenome analysis could deepen the understanding of the role of small intestinal microbiota in NSAID enteropathy, and maybe provide some specific microbiota that can be used in the initial diagnosis.

In conclusion, the microbial community is a novel field still to be explored even if the preliminary research was encouraging. The modulation of microbiota will provide a new strategy in NSAID enteropathy prevention.

## Author Contributions

XW, QT, HH, WZ, ML, DC, YG, and HC drafted the manuscript. HC, JH, YL, and BW were involved in study supervision. All the authors were involved in literature review and provided critical revision of the manuscript for important intellectual content. All authors contributed to the article and approved the submitted version.

## Funding

This work was supported by the grants from the National Natural Science Foundation of China (82070545 and 81970477) and the Key Project of Science and Technology Pillar Program of Tianjin (20YFZCSY00020).

## Conflict of Interest

The authors declare that the research was conducted in the absence of any commercial or financial relationships that could be construed as a potential conflict of interest.

## Abbreviations

NSAIDs, non-steroidal anti-inflammatory drugs; PG, prostaglandins; PPI, proton pump inhibitors; LPS, lipopolysaccharide; NF-κB, nuclear factor-kappa B; IL, interleukin; TNF, tumor necrosis factor; HMGB1, high mobility group box 1; iNOS, inducible nitric oxide synthase; NLRs, nucleotide-binding and oligomerization domain-like receptors; FMT, fecal microbial transplantation; NO, nitric oxide; DNA, desoxyribonucleic acid; nNOS, neuronal nitric oxide synthase.
